# Development of the Brief Ageing Perceptions Questionnaire (B-APQ): a confirmatory factor analysis approach to item reduction

**DOI:** 10.1186/1471-2318-14-44

**Published:** 2014-04-09

**Authors:** Eithne Sexton, Bellinda L King-Kallimanis, Karen Morgan, Hannah McGee

**Affiliations:** 1 Department of Psychology, Royal College of Surgeons in Ireland, Dublin, Ireland; 2 Department of Medical Gerontology, TILDA Project, Trinity College Dublin, Dublin, Ireland; 3 Perdana University–Royal College of Surgeons in Ireland, Kuala Lumpur, Malaysia

**Keywords:** Ageing perceptions, Self-perceptions, Self-regulation, Confirmatory factor analysis, Item reduction

## Abstract

**Background:**

This paper aimed to develop a short version of the 32-item Ageing Perceptions Questionnaire (APQ), a multi-dimensional measure based on Leventhal's self-regulation model. Ageing perceptions are a key area of interest for large-scale surveys of ageing populations. As these studies capture a broad range of health and social variables, included instruments need to be as concise as possible.

**Methods:**

Data from the Irish Longitudinal Study of Ageing (TILDA), a representative sample of community-dwelling individuals aged 50+ (n = 6,718), was used to revise the scale. Items for exclusion were identified by examining conceptual content, descriptive statistics, and by detecting sources of poor model fit using confirmatory factor analysis (CFA). Potential combinations of dimensions were also tested using CFA. Finally, we identified any dimensions that could be excluded without limiting the conceptual coverage and coherence of the scale. Model modifications were done sequentially and with regard to theoretical considerations. Internal consistency and construct validity of the concise scale were compared with the longer version.

**Results:**

Initially, 11 items were excluded on the basis of conceptual and empirical overlap with other items. CFA indicated that the negative-control and negative-consequences dimensions could be combined, allowing us to exclude a further item from this dimension. The 5-item timeline-cyclical dimension was also excluded, as it was less well-established conceptually and empirically than the other dimensions. The final 17-item, 5-dimension model was consistent with the original conceptual model and fit the data well (chi-sq = 1433.54, df(109), p < 0.01, RMSEA = 0.04, CFI = 0.97, TLI = 0.96).

**Conclusions:**

The Brief-APQ (B-APQ) is a concise, multi-dimensional measure of ageing perceptions, which is psychometrically valid for use with the Irish population aged 50+. The concise version preserved the internal consistency and construct validity of the original. Its brevity makes it particularly suitable for use with large-scale adult population surveys. The psychometric analysis supports the application of the self-regulation model to ageing perceptions, but also the existence of distinct "physical decline" and "ongoing development" dimensions of perceptions.

## Background

Older adults' self-perceptions of ageing have been shown to be important predictors of physical health and mortality [1,2], as well as key well-being outcomes such as quality of life (QoL) [3,4]. It is thought that these beliefs are shaped throughout the life-course, influenced by experience and by broader societal attitudes to ageing, and can influence outcomes via behavioural [5], psychological and potentially physiological pathways [1,2].

Two distinct dimensions to ageing perceptions have been identified – positive perceptions of ageing as ongoing personal growth and development, and negative perceptions of ageing as a time of physical decline [6]. These dimensions are examined in most of the studies cited above, with positive perceptions leading to better outcomes, and negative perceptions leading to worse [1,2,4].

On the other hand, it is possible that ageing self-perceptions are multi-dimensional, as people build a complex schema to make sense of the multi-faceted ageing process [6,7]. Barker et al developed the Ageing Perceptions Questionnaire (APQ), a multi-dimensional measure of ageing perceptions, drawing on Leventhal's self-regulation model of health and illness. The self-regulation model conceptualises illness as a stressor, with people's perceptions of an illness shaping how they respond to that illness, and which ultimately determines outcomes, including morbidity and mortality [8,9]. Illness perceptions are assumed to have diverse content, including time-line (duration or course of the illness), consequences (outcomes of illness such as disability or death), and control (the extent to which an illness can be cured or managed). The APQ applies this framework to perceptions of ageing as a potential stressor, positing seven dimensions. *Timeline-chronic* and *timeline-cyclical* refer to perceptions of the course of the ageing process as chronic (constant awareness of ageing) and cyclical (variations in awareness of ageing). *Consequences* relates to beliefs about the impact of ageing, and has negative and positive sub-dimensions. *Control* relates to beliefs about how much control a person has over aspects of ageing, both negative and positive. Finally, *emotional representations* refers to negative emotional responses to ageing, including anxiety, depression and worry.

Focus groups were used for initial content validation of the APQ [7], followed by testing of psychometric properties with a small sample of community dwellers aged 65+. Further evaluation with a large, population sample found that the 32-item questionnaire had good psychometric properties. Ageing perceptions are a key topic of concern of large scale surveys of ageing, given the evidence outlined above. However, such surveys require concise instruments, to allow the use of multiple measures and minimise respondent burden [10]. It is common practice to develop concise or brief versions of measures of health perceptions [11], as well as other psychological measures, such as QoL [12].

This paper seeks to develop a brief version of the APQ, with capacity to differentiate multiple dimensions of perceptions, while being sufficiently concise for inclusion in large-scale surveys with multiple measures, including future waves of the Irish Longitudinal Study of Ageing (TILDA). We aimed to shorten the scale using two strategies: 1) remove items characterised by empirical and conceptual overlap; 2) identify combinations of sub-dimensions that are psychometrically and conceptually valid. We hypothesised four potential combinations of sub-dimensions:

1. Negative and positive control sub-dimensions as an overall control factor

2. Negative and positive consequence sub-dimensions as an overall consequence factor

3. Negative consequence and control sub-dimensions as an overall negative perceptions factor

4. Positive consequence and control sub-dimensions as an overall positive perceptions factor

## Methods

### Data

The Irish Longitudinal Study of Ageing (TILDA) is a representative sample of community-dwelling individuals aged 50+ in the Republic of Ireland. Geographical clusters were selected at random, followed by households within each cluster. Any household members aged 50+, and their partners, were invited to participate. This study utilized Wave 1 data, collected between October 2009 and July 2011, with an estimated household response rate of 62% (n = 8,175). Data collection comprised a computer-assisted home interview, conducted by trained field workers, a self-completion questionnaire and a nurse-led health assessment. The survey captured a range of social, health, psychological and economic variables. Further sampling details are provided by Kearney et al [13]. Of the total sample, 6,718 completed at least half the items in the self-completion APQ scale, and were included in our analysis.

## Ethics

Ethical approval for TILDA was obtained from the Trinity College Dublin Research Ethics Committee. All participants provided written, informed consent.

### Measures

The APQ measure comprises 32 Likert scale items that represent seven ageing perception domains: timeline-chronic (items 1-5), timeline-cyclical (items 27, 28, 30-32), consequences-positive (items 6-8), consequences-negative (items 16–20), control-positive (items 10-12, 14, 15), control-negative (items 21-24) and emotional-representations (items 9, 13, 25, 26, 29). Participants rate their agreement with a series of statements – for example “*I am always aware of my age*” or “*As I get older I can take part in fewer activities*” with a five-point response scale with the options: Strongly Disagree, Disagree, Neither Agree nor Disagree, Agree, Strongly Agree. The negative control and consequences scales were reverse coded, so that higher scores indicated more positive perceptions of aging.

Theoretically relevant constructs were measured to test convergent and discriminant validity. Physical limitations was measured by number of difficulties with basic mobility activities (e.g. *walking 100 metres, sitting for about two hours*), from a list of 11 activities. Depressive symptoms was measured by score on the Centre for Epidemiological Studies Depression scale (CES-D) [14], which is intended for use with the general population. QoL was measured using two dimensions of the CASP-R12 – control/autonomy, the extent to which a person has control over their life, free from outside interference; and self-realisation/pleasure, the extent to which life is personally fulfilling and enjoyable [15]. Age in years, highest level of education completed (primary/secondary/third level) and sex were included as covariates.

### Statistical analysis

Following Goetz et al [10], we used a rigorous approach to scale revision, using confirmatory factor analysis (CFA) in conjunction with substantive considerations; producing a shorter scale while preserving the psychometric properties and conceptual model of the longer version. The analysis consisted of four broad steps. In Step 1, we tested the existing measurement model using CFA. Step 2 involved removing items with both empirical and conceptual overlap with other items in the scale. Step 3 involved final testing of the four alternative combinations of sub-dimensions. Finally, in Step 4 models were assessed to determine whether we had removed a sufficient number of items. If not, we removed one or more dimensions that were considered conceptually less relevant or unnecessary. Model revision was done sequentially, with alternative re-specifications and item composition considered at each step, seeking to ensure adequate overall conceptual coverage and coherence, as well as psychometric validity.

Mplus (Version 6) was used for CFA. Descriptive statistics, including response proportions for each item, and inter-item correlations were generated and inspected. The maximum-likelihood estimator with robust standard errors (MLR) estimator was used, due to high kurtosis (>3) among some items. Although the items were ordinal, response frequencies were not particularly skewed (see Table 1), and five response options were deemed sufficient to treat the data as continuous [16]. Missing item scores were handled using the Full Information Maximum Likelihood (FIML) approach, which is analogous to using the observed item responses to predict the missing values.

**Table 1:** Ageing perception questionnaire: item content, response frequencies and reasons for item exclusion

**APQ items**	**Strongly disagree**	**Disagree**	**Neutral**	**Agree**	**Strongly agree**	**Reason for exclusion**
*Timeline-chronic*						
1. I am conscious of getting older all the time	14%	25%	25%	31%	5%	Excluded due to overlap with Item 4 (r = 0.67, MI = 425.6, EPC = 0.33)
2. I am always aware of my age	13%	28%	20%	34%	5%	Excluded due to overlap with Item 1 (r = 0.73, MI = 294, EPC = 0.33)
**3. I always classify myself as old**	30%	43%	15%	10%	2%	**Retained**
**4. I am always aware of the fact that I am getting older**	12%	24%	18%	42%	4%	**Retained**
**5. I feel my age in everything that I do**	25%	45%	16%	12%	2%	**Retained**
*Consequences-positive*						
**6. As I get older I get wiser**	3%	10%	23%	52%	12%	**Retained**
**7. As I get older I continue to grow as a person**	2%	5%	19%	60%	14%	**Retained**
**8. As I get older I appreciate things more**	2%	3%	9%	65%	21%	**Retained**
*Control-positive*						
**10. The quality of my social life in later years depends on me**	2%	5%	10%	66%	17%	**Retained**
**11. The quality of my relationships with others in later life depends on me**	2%	4%	10%	68%	17%	**Retained**
**12. Whether I continue living life to the full depends on me**	2%	4%	8%	67%	20%	**Retained**
14. As I get older there is much that I can do to maintain my independence	2%	4%	10%	67%	17%	Excluded due to overlap with Item 12 (r = 0.56, MI = 174.495, EPC = 0.19)
15. Whether getting older has positive sides to it depends on me	1%	3%	10%	68%	18%	Excluded due to overlap with Item 14 (r = 0.73, MI = 1328.86, EPC = 0.51)
*Consequences-negative*						
16. Getting older restricts the things that I can do	5%	47%	18%	24%	7%	Excluded due to overlap with item 19 (r = 0.70; MI = 170.4; EPC = 0.20)
**17. Getting older makes me less independent**	3%	26%	18%	42%	10%	**Retained**
18. Getting older makes everything a lot harder for me	3%	23%	24%	41%	9%	Excluded due to overlap with item 17 (r = 0.77; MI = 107.25, EPC = 0.22)
**19. As I get older I can take part in fewer activities**	4%	42%	19%	30%	6%	**Retained**
**20. As I get older I do not cope well with problems that arise**	3%	23%	19%	45%	10%	**Retained**
*Control-negative*						
**21. Slowing down with age is not something that I can control**	5%	52%	17%	22%	4%	**Retained**
22. How mobile I am in later life is not up to me	5%	31%	15%	42%	8%	Excluded due to overlap with Item 24 (r = 0.61, MI = 828.570, EPC = 0.4)
23. I have no control over whether I lose vitality or zest for life as I age	3%	23%	15%	50%	10%	Excluded due to overlap with item 24 (r = 0.80, MI = 128.06, EPC = 0.45)
**24. I have no control over the effects which getting older has on my social life**	3%	22%	15%	51%	9%	**Retained**
*Timeline-cyclical*						
27. I go through cycles in which my experience of ageing gets better and worse	9%	30%	32%	27%	2%	Excluded due to dropped dimension
28. My awareness of getting older comes and goes in cycles	8%	27%	23%	40%	2%	Excluded due to overlap with Item 27 (r = 0.88, MI = 1181.31 , EPC = 0.5)
30. I go through phases of feeling old	15%	39%	19%	26%	2%	Excluded due to dropped dimension
31. My awareness of getting older changes a great deal from day to day	14%	43%	22%	20%	2%	Excluded due to dropped dimension
32. I go through phases of viewing myself as being old	18%	43%	17%	20%	2%	Excluded at Step 2 due to overlap with several items, e.g. item 30 (r = 0.77, MI = 61.87, EPC = 1.7)
*Emotional representations*						
**9. I get depressed when I think about how ageing might affect the things that I can do**	18%	39%	21%	18%	4%	**Retained**
13. I get depressed when I think about the effect getting older might have on my social life	19%	45%	21%	12%	3%	Excluded due to overlap with item 9 (r = 0.69, MI = 355.849, EPC = 0.28)
25. I get depressed when I think about getting older	20%	51%	17%	10%	2%	Excluded due to conceptual overlap with item 9, weak empirical overlap
**26. I worry about the effects that getting older may have on my relationships with others**	16%	52%	17%	13%	2%	**Retained**
**29. I feel angry when I think about getting older**	25%	55%	14%	5%	2%	**Retained**

Legend: Items retained in the final scale are in bold. MI = Modification Index, EPC = Expected Parameter Change, r = correlation coefficient.

Overall model fit was assessed using the adjusted chi-square test statistic in conjunction with a range of alternative fit indices, as recommended by Brown [17]. The root mean square error of approximation (RMSEA) and its 90% confidence interval were used, with values less than or equal to 0.05 indicating ‘close’ fit [17]. Two comparative fit indices were used – the comparative fit index (CFI) and the Tucker-Lewis index (TLI); in each case values > 0.95 indicated good fit [18]. While these cut-offs are best interpreted as rules-of-thumb rather than golden rules [19], they are useful indicators, in conjunction with conceptual considerations, of potential for model improvement.

#### ***Step 1: test the existing model***

The existing APQ measurement model was tested using CFA, in order to provide a baseline model to compare any revised models. This model had seven factors that were represented by 32 items.

#### ***Step 2: identify items for removal***

A list of candidate items for removal was created based on large inter-item correlations and conceptual overlap with other items. We also identified over-lapping item pairs through examination of modification indices (MI) (>10), expected parameter change (EPC) values >0.2, and large standardised residual covariances (>0.2). While these cut-points were used as a guide, we focussed primarily on the relative size of these indicators to inform choices around item retention and removal, in conjunction with changes in model fit following item removal, and the overall conceptual coverage of the scale in relation to the physical, social and psychological aspects of ageing. We ensured that each sub-scale retained three items for model identification purposes.

#### ***Step 3: test dimension combinations***

With items contributing to poor fit removed, we used CFA to test a series of models including the hypothesized dimension combinations. For the negative and positive sub-dimensions, we tested models which included residual covariances for either negative or positive items, to take account of any method effect arising from the direction of wording in the items [20]. Where a combination of dimensions displayed good psychometric properties, we re-tested alternative item sets, including those removed in Step 2, to ensure optimal conceptual coverage and psychometric validity.

#### ***Step 4: identify dimensions for removal***

If the previous strategies had not succeeded in shortening the scale sufficiently (removing approximately half the items), we considered which of the seven dimensions could be tenably removed without violating the psychometric validity, or conceptual coherence and coverage of the scale.

#### ***Comparison of internal consistency and construct validity***

Once the shortened scale was developed, the internal consistency and construct validity (convergent and discriminant validity) of the original scale and the shortened version were compared to ensure that psychometric properties were preserved. The internal consistency of each of the sub-scales was examined using Cronbach's alpha [21], and compared across the two versions. Construct validity was initially assessed by comparing correlations between the sub-scales.

Convergent validity was assessed by examining associations with theoretically relevant constructs. We hypothesised that more positive perceptions of ageing would be associated with lower physical limitations and depression, and improved QoL. Discriminant validity was assessed by comparing associations across different measures. We hypothesised that ageing perceptions would have a weaker association with physical limitations than QoL, on the basis that subjective appraisals are more influential on perceptions than objective health states. We hypothesised that the control perception dimensions would be more strongly associated with the control/autonomy dimension of QoL, relative to the other constructs; and that the emotional representations dimension would be more strongly associated with depression, relative to the other constructs. We also predicted that consequence- positive would be more strongly related to the self-realisation/pleasure dimension of QoL, which captures the extent to which life is enjoyable and personally fulfilling, relative to the other constructs.

These associations were tested using linear regression, adjusted for age, sex and education. Cases with missing items on the original version of each dimension were excluded, to ensure that the longer and shorter versions were tested on the same sample. Stata 11.0 was used to generate Cronbach's alpha and correlations, and for the regression analysis.

## Results

### Descriptive statistics

Of the 6,718 respondents, ten percent were missing on one item, with approximately seven percent missing on more than one item. These cases were included in the analysis using Full Information Maximum Likelihood (FIML) estimation (see Methods). Item 3 had the highest level of missingness, at 2.1%. Table 1 displays the item content and response frequencies.

Around 54% of the sample was female, with a median age of 62 (IQR 56-70 years). Almost one third of the sample had third-level education (32%), with 41% having secondary level and 27% primary or less. Almost half (46%) rated their health as very good or excellent, with 22% rating their health as fair or poor.

### Step 1: testing the established measurement model

The measurement model for the existing 32 item, 7 dimension scale did not have good fit to the data (Model 1.1, Table 2). While the RMSEA was within the acceptable range, the TLI and CFI were outside the range commonly accepted as good fit for these indices. Large modification indices and EPC values indicated that several items were closely related, or loaded on to more than one dimension of the scale.

**Table 2:** Model fit statistics at each step of model revision

**Model**	**Description**	**Chi-Square**	**df**	**RMSEA**	**90% CI**		**CFI**	**TLI**
	**Step 1: Test established model**							
1.1	7 Factors, 32 items	10056.02	444	0.057	0.056	0.058	0.837	0.818
	**Step 2: Remove Items**							
2.1	7 Factors, 21 items	1892.69	168	0.039	0.038	0.041	0.968	0.961
	**Step 3: Combine sub-dimensions**							
3.1	6 Factors, 21 Items	6197.67	171	0.072	0.071	0.074	0.890	0.865
	Consequence dimensions combined, Residual Covariances for Negative Items							
3.2	6 Factors, 21 Items	2593.06	171	0.046	0.044	0.047	0.956	0.946
	Consequence dimensions combined, Residual Covariances for Positive Items							
3.3	6 Factors, 21 Items	4664.29	171	0.063	0.061	0.064	0.918	0.899
	Control dimensions combined, Residual Covariances for Negative Items							
3.4	6 Factors, 21 Items	2532.91	171	0.045	0.044	0.047	0.957	0.947
	**Control** dimensions combined, Residual Covariances for Positive Items							
3.5	6 Factors, 21 Items Negative Control and Consequence items combined	2920.59	174	0.048	0.047	0.050	0.950	0.939
3.6	6 Factors, 21 Items Positive Control and Consequence items combined	6423.66	174	0.073	0.072	0.075	0.886	0.862
3.7	6 Factors, 20 items	1882.74	155	0.041	0.039	0.042	0.967	0.959
	Model 3.5 with Item 22 removed							
	**Step 4: Remove Sub-dimensions**							
4.1	5 Factors, 17 items	1433.54	109	0.043	0.041	0.045	0.968	0.960
	Model 3.7 with Timeline-Cyclical Removed							

Legend: RMSEA = Root Mean Square Error of Approximation; TLI = Tucker-Lewis Index; CFI = Comparative Fit Index.

### Step 2: item removal

Inspection of modification indices, residual variances and EPCs, in conjunction with substantive content, identified a number of items that had considerable empirical and conceptual overlap with other items. Table 1 displays which items were removed and the reasons for exclusion. For some items, for example item 14 "*As I get older there is much I can do to maintain my independence*", modification indices indicated that the item loaded on to several other dimensions, indicating poor conceptual specificity. Some items were removed for primarily conceptual reasons, for example, item 25 "*I get depressed when I think about getting older*" was removed to preserve conceptual coverage of emotional representations by retaining items covering depression, worry and anger.

In total, we removed 11 items from 6 dimensions, leaving each dimension with 3 items. No items were removed from the consequences-positive dimension as there were only three items in the original version. Removal of these poorly fitting items was associated with improved the fit of the measurement model (Model 2.1, Table 2). While the chi-sq statistic did not indicate good fit (chi-sq = 1862.69, df(168), p < 0.01) this index is particularly sensitive to large samples, and the remaining statistics were within the boundaries of good fit (RMSEA = 0.04, CFI = 0.97, TLI = 0.96).

### Step 3: dimension combination

Initial combination of negative and positive consequence dimensions led to a poor fitting model. We decided to test models including residual covariances for negative items (Model 3.1) and positive items (Model 3.2) to control for a method effect. While Model 3.2 appeared to display good fit, both models had unacceptable factor loadings for either the negative or positive items (<0.2), failing to support an underlying consequences factor. The results were similar for the combination of the negative and positive control dimensions, with broadly acceptable overall fit but low factor loadings for items in one or other of the sub-dimensions. Many of the inter-item correlations between negative and positive items were low (<0.3).

Combination of negative control and negative consequence items did result in a model with good fit and acceptable factor loadings (Model 3.5). Combination of positive control and consequence items, however, did not result in a model with good fit (Model 3.6). Once we combined the negative control and consequence items, it was possible to remove a further item from one of these dimensions. Item 22 "*How mobile I am in later life is not up to me*" had considerable empirical overlap with item 21 "*Slowing down with age is not something that I can control*" and item 24 "I *have no control over the effects which getting older has on my social life*" (see Table 1). Removal of item 22 resulted in a 6 factor, 20 item model with good fit to the data (Model 3.7, Table 2).

#### ***Step 4: dimension removal***

Finally, we considered whether all of the dimensions were essential to the theoretical coverage of the measure. As Barker et al [7] note, the time-line cyclical dimension of the self-regulation model has a weaker empirical evidence base than the timeline-chronic sub-dimension. In their own study, scores on this dimension did not independently predict either depression or disability. We decided to remove this sub-dimension, while retaining timeline-chronic to ensure that the scale still captured timeline perceptions of ageing.

**Figure 1:**
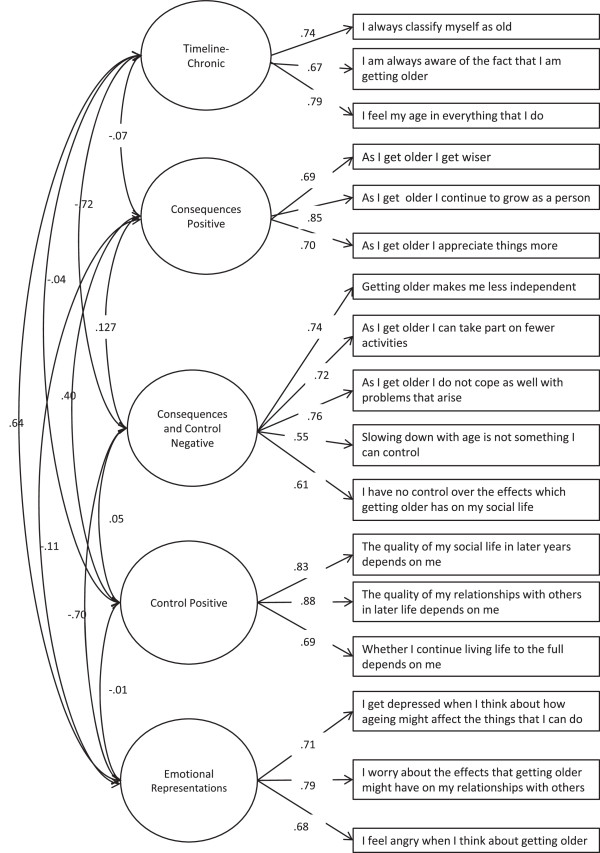
Unstandardised Parameters for Model 4.1.

This final model (4.1, Table 2) displayed excellent fit to the data, (chi-sq = 1433.54, df(109), p < 0.01, RMSEA = 0.04, CFI = 0.97, TLI = 0.96). Retained items are displayed in Table 1, while unstandardised parameter estimates for Model 4.1 are displayed in Figure 1. The final questionnaire is available online as Additional file 1.

#### ***Comparison of internal consistency and construct validity***

Table 3 displays the Cronbach's alpha for the sub-scales in the long and shortened versions. Internal consistency decreased for two sub-scales – timeline-chronic and emotional representations. This was not un-expected given the removal of redundant items. Cronbach's alpha was >0.7 for all sub-scales in the shortened version, indicating that internal consistency was preserved. The relationships between the dimensions were broadly preserved in the shortened version, with significant associations between dimensions in the same direction and of similar magnitude (see Table 4).

**Table 3:** Internal consistency of the sub-scales

**Long version**		**Shortened version**	
**Sub-scale**	**Cronbach's alpha**	**Sub-scale**	**Cronbach's alpha**
Time-line chronic	0.85	Time-line chronic	0.76
Time-line cyclical	0.87	Dropped	
Consequences positive	0.78	Consequences positive	0.78
Control positive	0.84	Control positive	0.84
Consequences negative	0.88	Consequences and control negative	0.81
Control negative	0.81		
Emotional representations	0.86	Emotional representations	0.75

**Table 4:** Correlations between APQ dimensions for the long and shortened versions

**Long version**	**1**	**2**	**3**	**4**	**5**	**6**
**Time-line chronic**						
**Time-line cyclical**	0.50					
**Consequences-positive**	-0.04*	-0.04**				
**Control-positive**	-0.08**	-0.05**	0.40**			
**Control-negative**	0.42**	0.35**	-0.10**	-0.18**		
**Consequences-negative**	0.56**	0.52**	-0.11**	-0.15**	0.57**	
**Emotional representations**	0.54**	0.69**	-0.11**	-0.11**	0.42**	0.55**
**Shortened version**	**1**		**2**	**3**	**4**	**5**
**Time-line chronic**						
**Consequences-positive**	-0.06**					
**Control-positive**	-0.07**		0.34**			
**Consequences and control-negative**	0.56**		-0.12**	-0.11**		
**Emotional representations**	0.50**		-0.10**	-0.05**	0.54**	

*p<0.05; **p<0.01.

Associations with relevant theoretical constructs were also preserved in the shorter version of the scale. As expected, views of ageing as a chronic process, negative perceptions of control over ageing, and of the consequences of ageing, and having negative emotional representations of the ageing process, were associated with more physical limitations, higher levels of depression and decreased QoL (see Table 5). In contrast, holding a positive view of the consequences of ageing, and having a sense of control over the ageing process, was associated with fewer physical limitations, lower levels of depression and increased QoL.

**Table 5:** Associations between dimensions of the APQ and physical limitations and depression and QoL, adjusted for age, sex and education, in the long and short versions

		**Physical limitations**			**Depressive symptoms**			**Self-Realisation/pleasure**			**Control/autonomy**	
	**N**	**B**	**Adjusted R**^**2**^	**N**	**B**	**Adjusted R**^**2**^	**N**	**B**	**Adjusted R**^**2**^	**N**	**B**	**Adjusted R**^**2**^
**Timeline-chronic (L)**	6,319	0.20**	0.15	6,226	0.23**	0.07	6,101	-0.28**	0.13	5,793	-0.37**	0.14
**Timeline-chronic (S)**		0.23**	0.16		0.24**	0.08	6,186	-0.29**	0.16		-0.39**	0.16
**Consequences-positive**	6,475	-0.03*	0.11	6,381	-0.08**	0.03	6,236	0.22**	0.06	5,914	0.11**	0.03
**Control-positive (L)**	6,451	-0.06**	0.12	6,358	-0.08**	0.03	6,229	0.17**	0.06	5,899	0.16**	0.04
**Control–positive (S)**		-0.04**	0.11		-0.03**	0.02		0.12**	0.03		0.11**	0.03
**Control-negative (L)**	6,225	0.18**	0.14	6,133	0.18**	0.05	6,021	-0.21**	0.17	5,710	-0.31**	0.10
**Consequences-negative (L)**		0.30**	0.19		0.29**	0.10		-0.33**	0.22		-0.53**	0.27
**Consequences and control-negative (S)**		0.27**	0.18		0.29**	0.09		-0.31**	0.24		-0.50**	0.23
**Emotional representations (L)**	6,414	0.18**	0.14	6,319	0.33**	0.13	6,202	-0.38**	0.15	5,885	-0.42**	0.19
**Emotional representations (S)**		0.18**	0.14		0.32**	0.12		-0.37**	0.14		-0.42**	0.19

*p < 0.05, **p < 0.01.

L = Long, S = Short.

Each APQ and B-APQ dimension was more strongly related to QoL than to physical limitations. Consequences-positive was more strongly associated with self-realisation/pleasure than with control/autonomy, depression, or physical limitations. Control negative was more strongly associated with control/autonomy relative to the other constructs. This was also true of consequences negative, and the combined negative control and consequences dimension.

Both the short and long version of control-positive had a relatively weak relationship with control/autonomy, contrary to our hypotheses. While emotional representations had a moderately strong relationship with depressive symptoms, it had similar associations with dimensions of QoL. Overall, however, the concise version displayed similar patterns of associations with other constructs, compared with the original version.

## Discussion

This paper described the development of a brief version of the APQ (B-APQ), using rigorous methodology based on both psychometric and conceptual criteria. The final B-APQ model had improved fit relative to the long version, and is consistent with the original conceptual model. It covers the key dimensions of the self-regulation model – timeline, control, consequences and emotional representations [8]. It has good conceptual coverage with the inclusion of items related to the physical, social and psychological aspects of ageing. Reliability, in terms of internal consistency, was preserved in the brief version. Convergent and discriminant validity were also preserved.

### Control and consequence dimensions

We found that the negative control and consequence items could be combined into an overall negative ageing perceptions dimension. Examining the item content, both of these dimensions captured loss of control and physical decline– e.g. "*Getting older makes me less independent*", "*Slowing down with age is not something that I can control*", consistent with our empirical findings that both dimensions were strongly associated with control/autonomy. This combined negative dimension appears to parallel a dimension of ageing perceptions identified in previous studies as "physical decline" [2,6].

On the other hand, the positive consequence items were more reflective of what Steverink et al [6] refer to as "ongoing development" - ageing as a period of continued learning and development of the self: for example, "*As I get older I get wiser*" and "*grow as a person*". This dimension was only weakly related to physical limitations, and most strongly related to self-realisation/pleasure. Our analysis therefore supports the idea of ageing perceptions as multi-directional – involving the "coexistence of gains and losses" [6].

The control-positive items, however, displayed low levels of empirical overlap with the control-negative items, despite apparent similarity in item content. For example, "*The quality of my social life in later years depends on me"* had an unexpectedly low correlation (<0.2) with "*I have no control over the effects which getting older has on my social life"*. Furthermore, this dimension was only weakly associated with control/autonomy. It is possible that the language used in the control-positive items – "*depends on me*", rather than "*control over*"– meant that something other than control beliefs was captured by these items. Further work is required to explore the meaning of this dimension.

### Ageing perceptions and the self-regulation model

Our analysis supported the existence of a timeline-chronic and an emotional representations dimension of ageing perceptions, providing support for the application of the self-regulation framework to ageing perceptions. The analysis of convergent and discriminant validity reported here provides some preliminary information on how these dimensions relate to other factors. Emotional representation was moderately associated with both depression and with QoL, suggesting that this dimension may capture both emotional and cognitive dimensions of ageing perceptions. Further work is required to understand the complex ways in which all of these dimensions are related to other constructs, and operate together and separately to influence outcomes.

Some aspects of the self-regulation model were more applicable to ageing perceptions than others. It was decided that the time-cyclical dimension did not add sufficient theoretical value to be retained in a concise version of the scale. This dimension has primarily been included in measures of illness perceptions to capture representations of cyclical or episodic illness such as allergies [22,8], and is therefore less relevant and applicable to ageing perceptions.

Similarly, two further components of the self-regulation model were not included in the version of the APQ included in TILDA: identity and cause, which relate to beliefs about what a stressor is, and what caused it [8]. The original APQ included these components by assessing perceptions of the extent to which any health problems were caused by ageing [7]. However, this part of the scale displayed a high ceiling effect, as a large proportion of older adults (c. 40%) who had experienced health-related changes, attributed these changes to ageing. This component of the scale was not included in TILDA.

### Limitations and further research

This study had a number of important limitations. Like the majority of these types of studies [10], resource and time constraints meant that judgements about conceptual content and coverage were made exclusively by the authors, with no independent lay or expert input. However, we clearly set out the rationale for our conceptual decisions, providing examples to justify our reasoning.

While we examined convergent and divergent validity, we did not examine predictive validity, as only one wave of data was available for analysis. In addition, it was decided that it was not possible or appropriate to attempt to define known-groups, as individuals with similar life circumstances may nevertheless hold diverse perceptions of the ageing process.

The use of a secondary dataset to revise the scale was a limitation as we did not choose measures for inclusion in the study. It would have been useful, for example, to assess the identity component of the APQ. Alternative measures of ageing perceptions, such as subjective age, would have been useful for establishing convergent validity. However, the use of secondary data was also a strength of the study, as psychometric analysis of the scale was based on the responses of a large, heterogeneous general sample of older people.

Finally, it was not possible to validate the shortened version in an independent sample of older people, and this would provide further support for the scale's validity [10]. It is planned that future waves of TILDA will use this shorter version, at least facilitating validation with a non-independent sample. Adoption of this measure by other studies of older populations would facilitate further validation of this measure.

## Conclusions

The B-APQ is a concise measure, which will make it particularly useful for large-scale surveys of ageing which tend to have intense competition for space among multiple measures of interest. It is likely to be useful in other research settings due to low respondent burden. It examines multiple dimensions of ageing perceptions, thus capturing the complexity of this experience among older people. Further work is required to examine how these dimensions differentially and longitudinally predict important health and psychosocial outcomes, which will be possible with successive waves of TILDA.

## Abbreviations

APQ: Ageing Perceptions Questionnaire; B-APQ: Brief-ageing perceptions questionnaire; CFA: Confirmatory factor analysis; CFI: Central fit index; EPC: Expected parameter change; FIML: Full information maximum likelihood; MI: Modification indices; MLR: Maximum likelihood robust; QoL: Quality of life; RMSEA: Root mean square error of approximation; TILDA: The irish longitudinal study of ageing; TLI: Tucker-lewis index.

## Competing interests

The authors declare that they have no competing interests.

## Authors’ contributions

ES participated in the design of the study, performed the statistical analysis and drafted the manuscript. BK conceived of the study and the study design, and participated in the statistical analysis and the drafting of the manuscript. HM and KM participated in the study design, contributed to the analysis and helped with the drafting of the manuscript. All authors read and approved the final manuscript.

## Pre-publication history

The pre-publication history for this paper can be accessed here:

http://www.biomedcentral.com/1471-2318/14/44/prepub

## Supplementary Material

Additional file 1 The Brief Ageing Perceptions Questionnaire. Click here for file
