# Supplementary material for: Development of the Brief Ageing Perceptions Questionnaire (B-APQ): a confirmatory factor analysis approach to item reduction
Source: BMC Geriatr. 2014 Apr 9;14:44. doi: 10.1186/1471-2318-14-44 (PMC4021231; doi:10.1186/1471-2318-14-44)
Supplement: Additional file 1 — The Brief Ageing Perceptions Questionnaire. [file 1471-2318-14-44-S1.pdf]

PLEASE TICK ONE BOX PER LINE WHICH SHOWS HOW YOU FEEL ABOUT EACH STATEMENT		STRONGLY DISAGREE	DISAGREE	NEITHER AGREE NOR DISAGREE	AGREE	STRONGLY AGREE
1	I always classify myself as old	☐	☐	☐	☐	☐
2	I am always aware of the fact that I am getting older	☐	☐	☐	☐	☐
3	I feel my age in everything that I do	☐	☐	☐	☐	☐
4	As I get older I get wiser	☐	☐	☐	☐	☐
5	As I get older I continue to grow as a person	☐	☐	☐	☐	☐
6	As I get older I appreciate things more	☐	☐	☐	☐	☐
7	I get depressed when I think about how ageing might affect the things that I can do	☐	☐	☐	☐	☐
8	The quality of my social life in later years depends on me	☐	☐	☐	☐	☐
9	The quality of my relationships with others in later life depends on me	☐	☐	☐	☐	☐
10	Whether I continue living life to the full depends on me	☐	☐	☐	☐	☐
11	Getting older makes me less independent	☐	☐	☐	☐	☐
12	As I get older I can take part in fewer activities	☐	☐	☐	☐	☐
13	As I get older I do not cope as well with problems that arise	☐	☐	☐	☐	☐
14	Slowing down with age is not something I can control	☐	☐	☐	☐	☐
15	I have no control over the effects which getting older has on my social life	☐	☐	☐	☐	☐

PLEASE TICK ONE BOX PER LINE WHICH SHOWS HOW YOU FEEL ABOUT EACH STATEMENT		STRONGLY AGREE	DISAGREE	NEITHER AGREE NOR DISAGREE	AGREE	STRONGLY AGREE
**16**	I worry about the effects that getting older may have on my relationships with others	☐	☐	☐	☐	☐
**17**	I feel angry when I think about getting older	☐	☐	☐	☐	☐

Sub-dimensions for Scoring:

Timeline-Chronic: Items 1, 2 and 3

Consequences-Positive: Items 4, 5 and 6

Emotional Representations: Items 7, 16 and 17

Consequences and Control Negative: Items 11, 12, 13, 14 and 15

Control-Positive: Items 8, 9 and 10
